# Forme rare du dysraphisme spinal fermé: la diastématomyélie: A rare form of closed spinal dysraphism: diastematomyelia

**DOI:** 10.11604/pamj.2017.28.317.14500

**Published:** 2017-12-27

**Authors:** Sadik Zbair, Asmaa Adnane, Kamilia Chbani, Siham Salam, Lahcen Ouzidane

**Affiliations:** 1 Université Hassan II, Faculté de Médecine et de Pharmacie, Service de Radiologie Pédiatrique, Hôpital Abderrahim Harouchi, CHU IBN Rochd, Casablanca, Maroc

**Keywords:** Diastématomyélie, dysraphisme spinal, anomalies du tube neural, Diastematomyelia, spinal dysraphism, neural tube defects

## Abstract

La diastématomyélie est une forme de dysraphisme spinal rare qui consiste en un dédoublement du canal vertébral et de son contenu. Deux types de diastématomyélie ont été décrits. Nous rapportons le cas d'un patient agé de 12 ans, de sexe masculin qui se présente pour une diminution de la force musculaire des membres inférieurs sans troubles sphinctériens associés. Le patient a bénéficié d'une imagerie par résonance magnétique (IRM) de la colonne vertébrale en séquence pondérée en T1 et T2, en coupe axiale, sagittale et coronale. L'IRM montre un aspect bifide de la moelle thoraco-lombaire en deux hémi-cordes sans éperon osseux séparant les deux hémi-moelles, compatible avec une diastématomyélie de type 1. Il s'y associe une moelle bas attachée avec cavité syringomyélique intéressant l'hémi moelle gauche et une lésion kystique biloculée au niveau de l'hémi moelle droite compatible avec un kyste neuro-enterique. L'IRM montre également la présence d'un défaut de fermeture de l'arc postérieur de D12 qui communique avec une poche sous cutanée en rapport avec un sinus dermique. La diastématomyélie est une anomalie rare de la colonne vertébrale qui peut être associée à d'autres malformations. La stratégie thérapeutique dépend essentiellement de la progression des signes cliniques (neurologiques) et des malformations associées.

## Abstract (en)

Diastematomyelia is a rare spinal dysraphism in which the spinal cord and its content are split. Two types of diastematomyelia have been described. We report the case of a 12 year old male patient presenting with reduced lower limb muscle strength without associated sphincteric disorders. The patient underwent axial, sagittal and coronal T1 and T2-weighted MRI sequence of the spine. MRI showed a bifid appearance of the bone marrow of thoracolumbar vertebrae in two hemi-cords without bone spur separating the two hemi-marrows, compatible with type 1 diastematomyelia. It was associated with low tethered spinal cord with syringomyelic cavity involving the left hemi-marrow and with biloculated fibrotic lesion at the level of the right hemi-marrow compatible with a neuroenteric cyst. MRI also showed incomplete closure of the posterior arch of D12 vertebra which communicated with a subcutaneous pocket in relation to a dermal sinus. Diastematomyelia is a rare abnormality of the spine which can be associated with other malformations. Therapeutic strategy essentially depends on the progression of the clinical signs (neurological) and of associated malformations.

## Introduction

La diastématomyélie ou la malformation du cordon médullaire divisé est une malformation médullaire rare caractérisée par une séparation sagittale plus ou moins étendue du canal vertébral et de son contenu pour donner un tube dédoublé. Elle est souvent associée à d'autres malformations. Nous allons décrire à travers ce cas l'aspect en imagerie de cette malformation.

## Patient et observation

**Figure 1:**
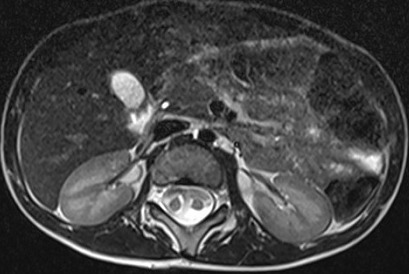
IRM médullaire en séquence axiale T2 montre un aspect bifide de la moelle en deux hémi-cordes sans éperon osseux

**Figure 2:**
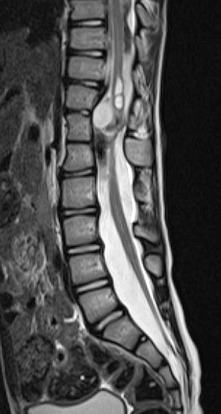
IRM médullaire en séquence sagittale T2 montre une cavité syringomyélique associée à un élargissement du canal médullaire et moelle bas attachée

**Figure 3:**
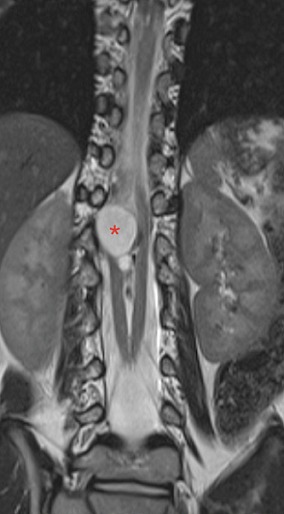
IRM médullaire en séquence coronale T2 montre un aspect bifide de la moelle avec kyste biloculé de l’hémi-corde droite compatible avec un kyste neuro-entérique (astérix)

**Figure 4:**
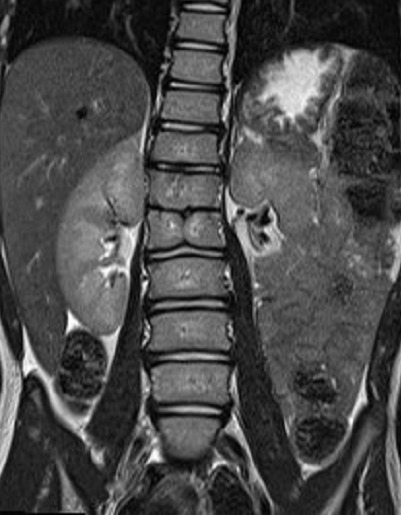
IRM médullaire en séquence coronale T1 montre une anomalie vertébrale somatique en aile de papillon

Un garçon agé de 12 ans, se présente pour une diminution de la force musculaire des membres inférieurs sans troubles sphinctériens associés. L'examen clinique montre une tuméfaction rénitente de la région lombaire avec touffe de cheveux sans signe de scoliose apparente. Le patient a bénéficié d'une imagerie par résonance magnétique (IRM) de la colonne vertébrale en séquence pondérée en T1 et T2, en coupe axiale, sagittale et coronale (Figure 1, Figure 2, Figure 3). L'IRM montre la présence d'un aspect bifide de la moelle thoraco-lombaire en deux hémi-cordes (Figure 1), sur une hauteur de 4 vertèbres entre D11 et L3 (Figure 3) sans éperon osseux séparant les deux hémi-moelles. Il s'y associe une cavité syringomyélique étendue sur 15 mm en regard de D11 au niveau de l'hémi moelle gauche (Figure 3). L'imagerie décèle également la présence d'une lésion kystique biloculée au niveau de l'hémi-moelle droite (asterisk:* Figure 1) mesurant 17 x 21 x 26mm siège de calcifications pariétales et non rehaussée après injection de gadolinium compatible avec un kyste neuro-entérique (Figure 4). Elargissement du canal médullaire avec attachement bas situé de la moelle en regard de S3 (Figure 2). Défaut de fermeture de l'arc postérieur de D12 qui communique avec une poche sous cutanée en rapport avec un sinus dermique. On notait également la présence d'autres d'anomalies vertébrales (anomalies somatiques en aile de papillon) (Figure 4). Absence de signes de scoliose.

## Discussion

On distingue deux variétés des diastématomyélies: Le type I regroupe les diastématomyélies avec enveloppes arachnoïdiennes et durales communes. Cette forme est rarement symptomatique. Elle n'est pas associée à un éperon osseux, mais à de simples adhérences fibreuses. Le type II est représenté par la présence de doubles enveloppes arachnoïdiennes et durales. Les deux sacs duraux sont le plus souvent séparés par un éperon osseux ou cartilagineux central [1]. Dans notre cas la diastématomyélie est de type 1 avec un septum fibreux. L'emplacement de la lésion peut siéger à n'importe quel niveau de la colonne vertébrale, cependant elle est fréquemment décrite au niveau de la charnière thoraco-lombaire [2]. Elle s'accompagne d'anomalies de développement vertébral; une scoliose est associée dans 60 à 70% des cas. L'association à une myéloméningocèle, une syringomyélie, ou un kyste neuro-entérique est rapportée [3]. La détérioration sur le plan neurologique des patients suivis pour diastématomyélie est attribuée à la traction exercée sur le cordon qui est généralement bas attaché, ces contraintes sont exagérées par la présence d'une cloison osseuse, et ont tendance à s'aggraver avec la croissance. Ainsi des signes neurologiques liés à la division médullaire; hypotrophie d'un membre inférieur, pied bot, et vessie neurologique peuvent être observés [2]. Elle prédomine chez le sexe féminin, et s'associe à des manifestations cutanées retrouvées dans le cas du dysraphisme: touffe de cheveux, fossettes, hémangiomes, naevus ou lipomes [4]. L'IRM et la TDM ont une place prépondérante dans le diagnostic et l'extension de cette malformation en précisant le siège et l'étendue de la diastématomyélie, son caractère uni ou multifocale, la position du cône terminal et des racines de la queue de cheval par rapport à la fente médullaire ainsi que l'existence de malformations associées mieux visualisées en reconstruction 3D et éventuelles complications (scoliose sévère, cavité intramédullaire, souffrance médullaire). Les Radiographies standards peuvent montrer une ou plusieurs anomalies suivantes: scoliose (60%) spina bifida (85 à 100%), un élargissement du diamètre transverse du canal vertébral, des anomalies de segmentation vertébrale ou de fusion intersegmentaire ou un éperon ossifié intra canalaire (inconstant). Dans certains cas, la radiographie peut être aveugle suite à la présence de septum fibreux ou cartilagineux [2]. L'échographie quant à elle, permet à un temps précoce avant six mois de montrer un spina bifida avec éversion des lames et élargissement du canal vertébral, la présence de deux hémi-moelles disposées l'une à côté de l'autre, un éperon échogène séparant les deux hémi-moelles ou la dilatation du canal épendymaire [5]. Le diagnostic anténatal est possible à l'échographie fœtale à partir de 22 à 24 SA [6].

## Conclusion

La diastématomyélie est une anomalie rare de la colonne vertébrale qui peut être associée à d'autres malformations. Pour prévenir les dommages neurologiques irréversibles et progressifs de la diastématomyélie, le diagnostic précoce et le traitement approprié sont d'une importance primordiale. Un mauvais diagnostic peut entraîner de graves séquelles neurologiques.

## Conflits d’intérêts

Les auteurs ne déclarent aucun conflit d'intérêts.
